# Biomechanical and morphological changes produced by ionizing radiation on bone tissue surrounding dental implant

**DOI:** 10.1590/1678-7757-2020-0191

**Published:** 2020-09-28

**Authors:** Priscilla Barbosa Ferreira Soares, Carlos José Soares, Pedro Henrique Justino Oliveira Limirio, Vitor Carvalho Lara, Camilla Christian Gomes Moura, Darceny Zanetta-Barbosa

**Affiliations:** 1 Federal University of Uberlândia School of Dentistry Department of Periodontology and Implantology UberlândiaMG Brasil Federal University of Uberlândia, School of Dentistry, Department of Periodontology and Implantology, Uberlândia, MG, Brasil.; 2 Federal University of Uberlândia School of Dentistry Department of Operative Dentistry and Dental Materials UberlândiaMG Brasil Federal University of Uberlândia, School of Dentistry, Department of Operative Dentistry and Dental Materials, Uberlândia, MG, Brasil.; 3 Federal University of Triângulo Mineiro School of Medicine Department of Radiology UberabaMG Brasil Federal University of Triângulo Mineiro, School of Medicine, Department of Radiology, Uberaba, MG, Brasil.; 4 Federal University of Uberlândia School of Dentistry Department of Endodontics UberlândiaMG Brasil Federal University of Uberlândia, School of Dentistry, Department of Endodontics, Uberlândia, MG, Brasil.; 5 Federal University of Uberlândia School of Dentistry Department of Oral and Maxillofacial Surgery UberlândiaMG Brasil Federal University of Uberlândia, School of Dentistry, Department of Oral and Maxillofacial Surgery, Uberlândia, MG, Brasil.

**Keywords:** Bone, Bones, Dental implants, Radiation, ionizing, Biomechanical phenomena, X-ray microtomography

## Abstract

**Objective:**

This study analyzed the effect of ionizing radiation on bone microarchitecture and biomechanical properties in the bone tissue surrounding a dental implant.

**Methodology:**

Twenty rabbits received three dental morse taper junction implants: one in the left tibia and two in the right tibia. The animals were randomized into two groups: the nonirradiated group (control group) and the irradiated group, which received 30 Gy in a single dose 2 weeks after the implant procedure. Four weeks after the implant procedure, the animals were sacrificed, and the implant/bone specimens were used for each experiment. The specimens (n=10) of the right tibia were examined by microcomputed tomography to measure the cortical volume (CtV, mm^3^), cortical thickness (CtTh, mm) and porosity (CtPo, %). The other specimens (n=10) were examined by dynamic indentation to measure the elastic modulus (E, GPa) and Vickers hardness (VHN, N/mm^2^) in the bone. The specimens of the left tibia (n=10) were subjected to pull-out tests to calculate the failure load (N), displacement (mm) up to the failure point and interface stiffness (N/mm). In the irradiated group, two measurements were performed: close, at 1 mm surrounding the implant surface, and distant, at 2.5 mm from the external limit of the first measurement. Data were analyzed using one-way ANOVA, Tukey’s test and Student’s t-test (α=0.05).

**Results:**

The irradiated bone closer to the implant surface had lower elastic modulus (E), Vickers hardness (VHN), Ct.Th, and Ct.V values and a higher Ct.Po value than the bone distant to the implant (P<0.04). The irradiated bone that was distant from the implant surface had lower E, VHN, and Ct.Th values and a higher Ct.Po value than the nonirradiated bone (P<0.04). The nonirradiated bone had higher failure loads, displacements and stiffness values than the irradiated bone (P<0.02).

**Conclusion:**

Ionizing radiation in dental implants resulted in negative effects on the microarchitecture and biomechanical properties of bone tissue, mainly near the surface of the implant.

## Introduction

The life expectancy of the world population has increased, and consequently, the need for dental implants as part of oral rehabilitation did as well.^1^^,^^2^ The incidence of head and neck cancer has also increased, and radiotherapy may be indicated for patients with previously installed dental implants. Thus, clinicians are faced with the question of whether to remove or maintain osseointegrated implants before radiotherapy for head and neck cancer treatment. Several studies have been performed to evaluate implant survival in previously irradiated areas.^2^^–^^5^ However, few studies have evaluated the presence of osseointegrated implants in irradiated bone areas.^1^^,^^4^^,^^6^^,^^7^

The presence of titanium implants in irradiated areas can create a deleterious effect on bone tissue. Backscatter high-energy photons and electrons at the tissue-metal interface may also compromise bone repair.^5^^,^^8^^–^^10^ In addition, ionizing radiation induces persistent hypoxia in small blood vessels and decreases the activity and quantity of osteoblasts and osteocytes,^2^^,^^5^^,^^10^ which can increase the occurrence of osteoradionecrosis.^5^^,^^9^

These effects have hindered efforts to determine the best moment to install implants after irradiation.^2^^,^^4^ During head and neck tumour ablative surgery, dental implants can be installed in areas that need to be treated using radiotherapy.^1^^,^^4^^,^^6^^,^^7^ The stability of titanium implants in the osseointegration process is compromised by radiation in a dose-dependent manner.^11^^,^^12^ Additionally, there is no consensus regarding the impact of ionizing irradiation on the functionality or survival of the implant installed in the irradiation field. Notably, the effect of ionizing radiation is dose dependent.^11^^,^^12^ A single dose of 30 Gy has been demonstrated to be sufficient to cause a negative influence on bone/implant integrity in a rabbit study model.^11^^,^^12^

Radiotherapy is one of the most common treatments for head and neck cancer patients,^4^^,^^13^ and ionizing radiation can reduce bone healing capacity through the progressive fibrosis of blood vessels and soft tissue,^14^ loss of osteoblast function^15^ and damage to the collagen arrangement.^16^ These effects can also negatively influence bone/implant integration.^5^^,^^10^ Studies have been carried out to evaluate implant survival in previously irradiated areas,^2^^,^^3^^,^^5^ but there are limited data on the effect of backscattered radiation on the osseointegration process of implants placed before ionizing radiation.^1^^,^^4^^,^^5^^,^^7^^,^^8^ Although backscattering effects around the implant can be a problem for individuals with implant rehabilitation,^1^^,^^4^^,^^10^ the risk of radionecrosis is not significantly higher than that for postimplantation radiotherapy.^1^^,^^4^^,^^10^ Therefore, the aim of this study was to evaluate the effects of ionizing radiation on the rabbit bone surrounding an implant using microcomputed tomography (micro-CT) and biomechanical analysis.

## Methodology

The present preclinical *in vivo* study is reported according to the ARRIVE guidelines regarding all relevant items. The animal experimental protocol was approved by the Bioethics Committee for Animal Experimentation (CEUA #093/12) at the Universidade Federal de Uberlândia. This study followed the normative guidelines of the National Council for Animal Control and Experimentation (CONCEA), a subsidiary of the Ministry of Science, Technology and Innovation (MCTI; Law 11.794, 08/19/2008), Brazil. Twenty New Zealand white male rabbits that weighed between 3.0 and 3.5 kg and were 6 months of age were included in the study. All animals were acclimatized for 2 weeks before the experimental procedures. The animals were randomly and individually housed in standard cages containing bedding and nesting material at the ambient temperature of 20°C under controlled humidity and a 12-hour circadian rhythm. The diet consisted of standard laboratory pellets and water *ad libitum*. The animal caretakers were blinded to the experimental groups. The animals received three implants in their tibias (one in the left tibia and two in the right tibia) and were randomized into two groups (n=10): a nonirradiated group, in which the animals were not subjected to ionizing radiation, and an irradiated group, in which the animals received external irradiation of both tibias 2 weeks after the implant installation surgery.

### Surgical procedure

**Figure 1:**
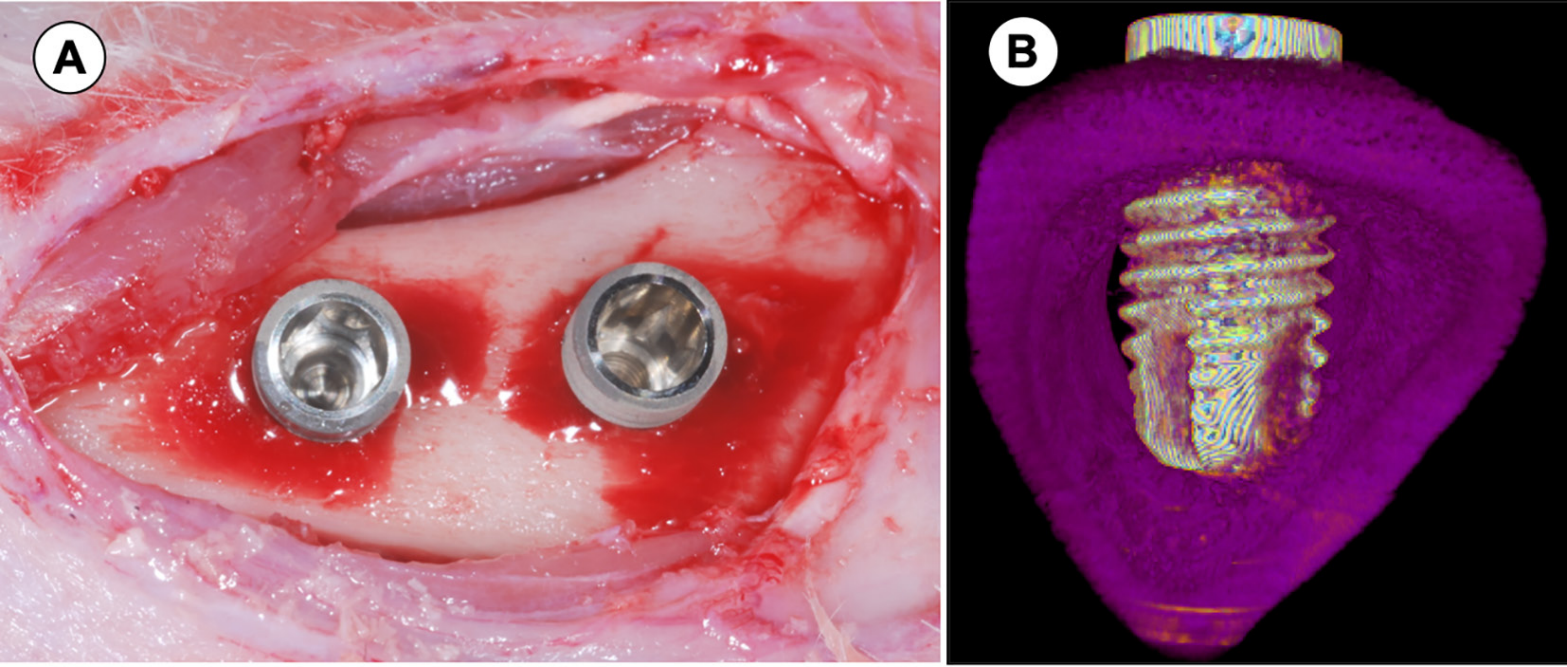
Implant installation on the rabbit tibia. A - Two implants installed on the right tibia with dissection of soft tissue and periosteum. B - Schematic 3D model of transverse tibia section showing the implant installed in cortical bone

The animals were fasted for twelve hours prior to surgery. For sterile preparation of the surgical site, the animal legs were shaved, and the tibia areas were cleaned with a 0.2% chlorhexidine solution (Rioquimica, São José do Rio Preto, SP, Brazil). The animals were anaesthetized through intramuscular injection with a combination of 0.25 mg ketamine/kg body weight (Ketamina Agener^®^; Agener União Ltda., São Paulo, SP, Brazil) and 0.5 mg xylazine/kg body weight (Rompum^®^ Bayer S.A. São Paulo, SP, Brazil). The anaesthesia was administered with 2% lidocaine and 1:100,000 epinephrine (Alphacaine^®^ 0.5 - 1 ml/site, DFL, Rio de Janeiro, RJ, Brazil) to reduce stimulation during surgery and to generate vasoconstriction. Incisions of 3 cm in length were made in both tibias. The soft tissue and periosteum were removed, and a sharp subperiosteal dissection exposed the proximal tibia. Grade 4 titanium dental implants and a morse taper junction, measuring 3.75 mm in diameter and 7.0 mm in length (Titamax Acqua CM, Neodent^®^, Curitiba, PR, Brazil), were inserted into each animal in the diaphysis region, which primarily contains cortical bone. One implant was installed in the left tibia and two in the right tibia, at a distance of 10 mm (Figure 1A) between the implants as measured by a periodontal probe. The implants were placed using a progressive sequence of drills under constant irrigation with 0.9% sodium saline solution according to the manufacturer’s instructions. All drilling procedures were performed at 1200 rpm, while considering the depth parameter based only on the rupture of one external cortical bone (Figure 1B). The soft tissues were sutured in separate layers using an interrupted suture (#5.0 nylon sutures Ethicon^®^: Johnson & Johnson Medical Ltd., Blue Ash, Ohio, United States). To prevent infection, daily intramuscular injections of cefazolin (Ourofino, São Paulo, SP, Brazil, 250 mg/kg) were given for 1 week. To prevent pain, a 0.3 mg/kg dose of the anti-inflammatory Meloxicam^®^ (Ourofino) was given. Each rabbit was caged individually at room temperature and received food and water. After 2 weeks of surgery, the animals were randomly divided into nonirradiated and irradiated groups.

### Irradiation protocol

After 2 weeks of implant installation, irradiation was performed on the irradiated group. During the irradiation sessions, animals in the irradiated group were maintained under sedation by intramuscular injection with a combination of 1.3 ml ketamine (100 mg/kg) and xylazine chlorate (7 mg/kg body weight). Both hind legs of each rabbit were subjected to irradiation using a single dose of 30 Gy.^11^^,^^12^^,^^17^ A 5-mm bolus was given to ensure full build-up. The tibia metaphysis region of the hind leg was the designated zone for irradiation. A single dose of radiation was delivered with a source–skin distance of 60 cm and a field measuring 15x15 cm with a direct electron beam of 6 MeV (Varian 600-C^®^ Varian Medical Systems Inc, Palo Alto, California, USA). The dose rate was 400 cGy/min. After irradiation, the veterinarian closely monitored the skin, hair, weight, and appetite of the rabbits for 2 weeks.

### Animal sacrifice and sample preparations

All animals were sacrificed 4 weeks after implant installation. The animals were anaesthetized with 2.5% thiopental and sacrificed with an intravenous injection of 19% potassium chloride (Ariston Chemical and Pharmaceutical Industry Ltda. São Paulo, SP, Brazil). The overlying soft tissues were removed, and the tibia were stored in plastic tubes containing phosphate-buffered saline solution and frozen at -20°C before testing. The implant installed in the left tibia was used for the pull-out test, one implant installed in the right tibia was used for the micro-CT analysis, and the other implant was used for the dynamic indentation test.

### Microcomputed tomography (micro-CT) analyses

The bone/implant samples (n=10) were scanned at an energy of 90 kV and an intensity of 278 mA with a resolution of 9 µm pixels using a Cu 0.1 mm filter (Skyscan-1272 X-ray microtomography; Bruker, Kontich, Belgium). The reconstructed 3D data sets were quantified using the CTAn automated image analysis system (Bruker). The volume of interest (VOI) for cortical analyses was selected around the implant and defined as a column from the implant axis with a radius of 1.5 mm within cortical bone, extending for a total of 200 slices. The implant was selected based on its threshold level, and this region was circumferentially expanded, creating a 0.55-mm zone around the implant. To compare the effect of metal on irradiation enhancement in the irradiated group, two measurements were performed on the same bone volume: close, at 1 mm surrounding the implant surface, and distant, at 2.5 mm from the external limit of the first measurement. The following microarchitecture parameters were analysed in the VOI images according to standard procedures:^18^^,^^19^ cortical volume (CtV, mm^3^), cortical thickness (CtTh, mm) and porosity (CtPo, %).

### Dynamic indentation test

**Figure 2:**
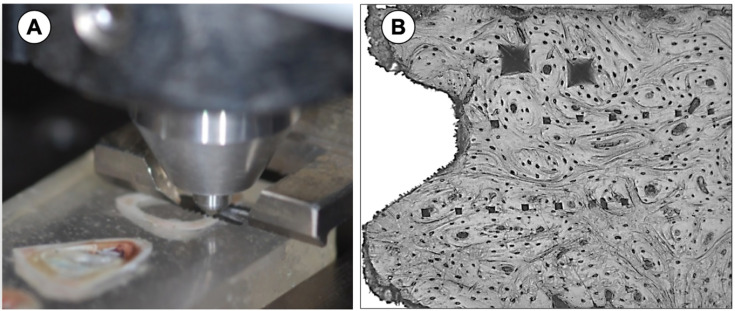
Dynamic Indentation test. A - Indentation moment in metallic device with the tibia embedded in polyester resin. B - Two indentations in a cortical bone close to the implant surface

The elastic modulus (E, GPa) and Vickers hardness (VHN, N/mm^2^) of the bone samples (n = 10) were assessed by using a microhardness dynamic indenter (CSM Micro-Hardness Tester; CSM Instruments, Peseux, Switzerland). The sample preparation and experimental protocol were performed as described previously by Soares, et al.^20^ (2014). The samples were embedded in polyester resin (Instrumental Instrumentos de Medição Ltda, São Paulo, SP, Brazil) using a metallic device (Metalon; Metalon Pooled Industries, Nova Iguaçu, RJ, Brazil) (Figure 2A). Using a Vickers indenter, seven continuous indentations were made with a 0.08 mm distance between each one (Figure 2B). Two measurements were performed on the same sample close and distant to the implant, following the measurements made in the micro-CT analysis. The indentation was carried out with controlled force, whereby the test load was increased or decreased at a constant speed ranging between 0 and 200 mN in 60-second intervals. The maximum force of 200 mN was held for five seconds. The load and penetration depth of the indenter were continuously measured during load-unload hysteresis. Universal hardness was defined as the applied force divided by the apparent area of the indentation at the maximum force. The measurements were expressed in VHN units by applying the conversion factor supplied by the manufacturer. The indentation modulus was calculated from the slope of the tangent of the indentation depth curve at the maximum force, which was comparable to the E of the bone structure.

### Pull-out test

The tibia/implant sample (n=10) was mounted in a customized device during the pull-out tests. The device was adjusted to align with the load cell. This mechanical test consisted of applying an increasing vertical force along the implant axis until the bone-implant interface was broken. A mechanical testing machine (EMIC DL 2000; EMIC, São José dos Pinhais, PR, Brazil) fitted with a calibrated load cell of 1000 N was used to perform the pull-out tests.^11^ The crosshead speed range was set to 1.0 mm/min. Data were graphed as force versus displacement, and the failure load (N), displacement (mm) up to the failure point and interface stiffness (N/mm) were also calculated from the graph.

### Statistical analysis

The CtV, CtTh, Ct.Po, E, VHN, and pull-out data were tested for normal distribution (Shapiro-Wilk, P>0.05) and equality of variances (Levene’s test), followed by parametric statistical tests. One-way analysis of variance (ANOVA) was performed for the Ct.V, Ct.Th, Ct.Po, E and VHN values. Tukey’s test was used for multiple comparisons. Student’s t-test was performed for the pull-out data. A *post hoc* test was performed to define the minimum difference in the parameters assessed in this study that would have been possible to detect by applying a power of 80%. All tests employed an α 0.05 significance level, and all analyses were carried out with the statistical package SigmaPlot version 13.1 (Systat Software Inc., San Jose, CA, USA).

## Results

### Micro-CT analysis – bone microarchitecture

The micro-CT results are shown in Table 1. The nonirradiated group had significantly higher Ct.V (P<0.022) and lower Ct.Po (P<0.002) values than the irradiated group close and distant to the implant. In the irradiated group, the Ct.V (P=0.032) and higher Ct.Po (P=0.025) values of the bone close to the implant were significantly lower than those of the bone distant to the implant. However, no significant difference was observed between groups in terms of the Ct.Th values (P=0.412).

**Table 1:** Mean and standard deviation values of cortical volume (Ct.V), cortical thickness (Ct.Th), and porosity (Ct.Po) measured by micro-CT analysis for nonirradiated and irradiated group close and distant from the implant surface

Groups		Ct.V (mm^3^)	Ct.Th (mm)	Ct.Po (%)
Nonirradiated animals group		6.9±0.3^A^	0.30±0.06^A^	65.9±1.4^A^
Irradiated animal group	Measured distant to the implant surface	6.5±0.3^B^	0.31±0.03^A^	68.4±1.1^B^
	Measured close to the implant surface	6.1±0.3^C^	0.33±0.03^A^	71.9±1.7^C^

Superscript letters represent significant difference within each morphological parameter, defined by Tukey test (P<0.05).

### Dynamic indentation test – E and VHN

The dynamic indentation test results are shown in Table 2. The bone tissue of the nonirradiated group had significantly higher E (P<0.001) and VHN values (P=0.001) than the bone tissue of the irradiated group, both close and distant to the implant. In the irradiated group, the E and VHN values of the bone distant to the implant were significantly higher than those of the bone close to the implant (P=0.034).

**Table 2:** Mean and standard deviation values of elastic modulus and Vickers hardness measured by dynamic indentation test for non-irradiated group and irradiated group close and distant from the implant surface

Groups		Elastic modulus (GPa)	Vickers hardness (N/mm^2^)
Nonirradiated animals group		20.8±3.2^A^	115.9±32.5^A^
Irradiated animals group	Measured distant to the implant surface	18.3±2.5^B^	91.5±32.0^B^
	Measured close to the implant surface	16.1±2.5^C^	69.7±27.2^C^

Superscript letters represent significant difference within each mechanical property, defined by Student’s t-test (P<0.05).

### Pull-out test – implant/bone structure stability

The pull-out test results are shown in Table 3. The nonirradiated group had significantly higher failure loads (P=0.002), higher displacements (P<0.001) and higher interface stiffness values (P=0.019) than the irradiated group.

**Table 3:** Mean and standard deviation values of failure load, displacement up to the failure point and interface stiffness measured by pull-out test for nonirradiated and irradiated group

Groups	Failure load (N)	Displacement (mm)	Stiffness (N/mm)
Nonirradiated animals group	406.7±51.8^A^	1.67±0.74^A^	339.8±89.4^A^
Irradiated implants group	321.4±89.4^B^	0.79±0.20^B^	287.9±64.6^B^

Superscript letters represent significant difference within each mechanical parameter, defined by Tukey test (P<0.05).

## Discussion

The results of this study showed that ionizing radiation decreases bone mass, compromising the biomechanical properties of bone around dental implants. Despite the limitations of using animals to mimic clinical situations, such studies are still essential for the design of future clinical studies that aim to validate clinical protocols. These findings may help to contribute to the establishment of predictable and successful treatment protocols for dental implant rehabilitation before radiotherapy in patients with head and neck cancer, as consistent information about the effect of backscattering radiation from titanium on the bone surface is lacking.

The rabbit tibia model used in this study is considered valid for evaluating biomechanical properties in relation to the osseointegration process after implant placement^21^. This animal possesses Havers systems similar to those found in humans^22^ and a thrice as fast bone turnover rate, allowing small periods of analysis of the osseointegration process^23^. An interval of 4 weeks was used between the placement of implants and the sacrifice of the animal to simulate a period of early osseointegration in humans that provides the basis for current treatment protocols.^18^^,^^24^^,^^25^ In addition, this study used a single dose of 30 Gy in 2 weeks after implant placement, aiming to impair bone healing,^12^^,^^15^ according to a previous study by Soares^25^ (2015). The single dose of 30 Gy radiation was also used in a rabbit study that demonstrated a low volume of newly formed bone between the labels, which suggested that the rate of bone formation is slow.^11^^,^^12^

The micro-CT results involved three microstructural parameters that are complementary and used to qualify cortical bone integrity and quality. The decrease in bone mass found in the irradiated groups of bone tissue both close and distant to the implant may have occurred due to the impairment of vascularization and osteoblast activity. Some studies have shown that ionizing radiation damages vascular endothelial cells, followed by occlusion and obliteration of some blood vessels, which may reduce the perfusion of osteogenic cells, mainly in the area of bone formation.^14^^,^^26^ Moreover, apoptosis is induced in osteoblasts exposed to irradiation, as they have higher radiosensitivity than other bone cells.^27^ Three-dimensional micro-CT analysis was used as this modality is recommended to quantify the bone matrix and to present results that are similar to those found in histomorphometric analyses.^28^ In addition, this study used biomechanical tests to determine the degree of bone-implant contact stability.

The lower values of failure load, displacement and interface stiffness measured in bone tissue close and distant to the implant in the irradiated group suggest that ionizing radiation damages the organic and mineral matrices. It is possible that irradiation affects the collagen arrangement, which decreases the mineralization process. The results confirmed the influence of irradiation on bone/implant integration, reducing the failure load, displacement and stiffness. When the implant is subjected to the pull-out test, the tensile force is transferred to the interface, showing that bone contact integrity is compromised by irradiation. Since the failure load was reduced, less displacement of the implant was necessary to cause rupture at the interface. Additionally, the calculated stiffness by the pull-out test is indirectly determined by the resistance imposed by the bone tissue to the implant removal, explaining the lower stiffness observed for the irradiated group.

Some studies have shown that irradiation increases plastic deformation in bone tissue by releasing free radicals via radiolysis of water molecules, degrading collagen molecules and restricting fibrillary sliding mechanisms,^29^^,^^30^ which affect the proper molecular arrangement for the biomineralization process to occur.^16^ In addition, irradiation may affect the activity of osteoblasts in terms of normal deposition and development of hydroxyapatite crystals from the inorganic matrix.^29^^,^^30^ The secondary effect caused by ionizing radiation is related to the implant composition, and we agree that the implant composition is decisive for the bone response. The grade 4 titanium implant had more of an effect on bone tissue than the titanium implant coated with hydroxyapatite that was subjected to ionizing radiation.^8^ This effect is more sensitive to the interface because this area is the highest dose enhancement.^9^^,^^31^

Furthermore, the lower values for E, VHN and bone mass volume when close to irradiated implants demonstrated that the deleterious effects of irradiation were more intensive in the region of bone-implant contact. A previous study^31^ examined the dose enhancement from scattered radiation at bone-dental implant interfaces and found a 21% maximum increase in the dose to alveolar mandibular bone in close proximity to the titanium. That study stated that a local overdose of 15 to 21% could cause a significant increase in the incidence of bone necrosis around osseointegrated titanium implants. Friedrich, et al.^31^ (2010) also reported that the presence of titanium dental implants in the field of irradiation caused osteoradionecrosis, corroborating the hypothesis of the backscattering effect of secondary electrons.

Our biomechanical findings and micro-CT analysis are supported by studies showing that the presence of titanium dental implants in the irradiation field induces a backscattering effect of secondary electrons, increasing the deleterious effects of irradiation on bone tissue around the implant.^5^^,^^15^ It has been very challenging for dentists to increase the success rate of dental implants in irradiated bone areas. Some human studies have indicated that exposure of bone to an irradiation dose exceeding 50 Gy impairs its ability to osseointegrate, increasing the failure risk of subsequent rehabilitation with a dental implant.^2^^,^^5^

This study has the same limitations as other studies, including that there was no load on the implants and that the implants were installed only in cortical bone. Most likely, the fatigue process of the loading process may intensify this influence. The use of a single dose of ionizing radiation in an animal research model can also be considered a limitation as the healing process can be intensified when the dose is fractionated by a systemic response. The results of this study cannot be directly extrapolated to clinical practice, but our findings may indicate a possible correlation with the irradiation response observed in humans.^15^ In patients with head and neck cancer that need to undergo radiotherapy, the observation of previously installed implants should be an important consideration.^7^^,^^8^ Given the lack of protocols that aim to address such situations, the irradiation field should be limited as much as possible to avoid implant areas, and patients need to return frequently to the dental office to analyse implant stability.

## Conclusion

Within the limitations of this *in vitro* study that tested the ionizing radiation over the pre-installed implants, the following conclusions can be drawn:

Irradiation decreased the failure load and displacement of implant when tested by pull-out test.

Irradiation decreased the mechanical properties, expressed by elastic modulus, Vickers hardness and stiffness of bone tissue around the implant.

Irradiation decreased the cortical volume and increased the porosity of bone around the implant.
